# The effect of depth data and upper limb impairment on lightweight monocular RGB human pose estimation models

**DOI:** 10.1186/s12938-025-01347-y

**Published:** 2025-02-07

**Authors:** Gloria-Edith Boudreault-Morales, Cesar Marquez-Chin, Xilin Liu, José Zariffa

**Affiliations:** 1 KITE Research Institute, Toronto Rehabilitation Institute – University Health Network, Toronto, ON Canada; 2 Institute of Biomedical Engineering, University of Toronto, Toronto, ON Canada; 3 Edward S. Rogers Sr. Department of Electrical and Computer Engineering, University of Toronto, Toronto, ON Canada; 4 Rehabilitation Sciences Institute, University of Toronto, Toronto, ON Canada

**Keywords:** Pose estimation, Depth data, Motion capture, Rehabilitation, Stroke

## Abstract

**Background and objectives:**

Markerless vision-based human pose estimation (HPE) is a promising avenue towards scalable data collection in rehabilitation. Deploying this technology will require self-contained systems able to process data efficiently and accurately. The aims of this work are to (1) Determine how depth data affects lightweight monocular red–green–blue (RGB) HPE performance (accuracy and speed), to inform sensor selection and (2) Validate HPE models using data from individuals with physical impairments.

**Methods:**

Two HPE models were investigated: Dite-HRNet and MobileHumanPose (capable of 2D and 3D HPE, respectively). The models were modified to include depth data as an input using three different fusion techniques: an early fusion method, a simple intermediate fusion method (using concatenation), and a complex intermediate fusion method (using specific fusion blocks, additional convolutional layers, and concatenation). All fusion techniques used RGB-D data, in contrast to the original models which only used RGB data. The models were trained, validated and tested using the CMU Panoptic and Human3.6 M data sets as well as a custom data set. The custom data set includes RGB-D and optical motion capture data of 15 uninjured and 12 post-stroke individuals, while they performed movements involving their upper limbs. HPE model performances were monitored through accuracy and computational efficiency. Evaluation metrics include Mean per Joint Position Error (MPJPE), Floating Point Operations (FLOPs) and frame rates (frames per second).

**Results:**

The early fusion architecture consistently delivered the lowest MPJPE in both 2D and 3D HPE cases while achieving similar FLOPs and frame rates to its RGB counterpart. These results were consistent regardless of the data used for training and testing the HPE models. Comparisons between the uninjured and stroke groups did not reveal a significant effect (all *p *values > 0.36) of motor impairment on the accuracy of any model.

**Conclusions:**

Including depth data using an early fusion architecture improves the accuracy–efficiency trade-off of the HPE model. HPE accuracy is not affected by the presence of physical impairments. These results suggest that using depth data with RGB data is beneficial to HPE, and that models trained with data collected from uninjured individuals can generalize to persons with physical impairments.

## Introduction

The development of new rehabilitation approaches requires appropriate measurement techniques to assess outcomes and personalize treatment. For example, in the rapidly evolving field of investigational neuromodulation therapies, quantifying movement performance is essential [1]. Motion tracking techniques are the most appropriate tool to quantify movement and obtain kinematic information.

Marker-based optical Motion Capture (MoCap) systems are the current motion tracking gold-standard due to their high accuracy [2, 3]. This type of system uses infrared multi-camera setups and physical markers, which require expensive dedicated instrumentation and is typically time-consuming to set up [4–6]. Data collection is constrained to the laboratory environment where the equipment is located, which limits the amount and variability of data collected [3, 7, 8]. On the other hand, informing and evaluating rehabilitation practices may require data to be collected in varied environments, such as hospitals, community clinics, and patient homes. To enable scalable data collection in rehabilitation, there is a need for low-cost and easy-to-use, yet accurate, accessible and consistent motion tracking techniques.

This need can be addressed using deep learning-based motion capture from video data, known as human pose estimation (HPE). HPE models have greatly benefited from advances in deep learning, such as convolutional neural networks (CNNs), which are very useful for extracting representative features from images [7, 9]. The development of techniques has been so thorough that the current 2D and 3D HPE state-of-the-art mostly consists of deep learning-based approaches [10]. Many studies have explored the use of HPE for rehabilitation-based applications [6, 11–13].

Tracking joint positions using HPE can enable kinematic analysis, which provides important information about how a movement is executed and its quality [14]. Since movements are typically performed in the 3D plane, 3D HPE models are better suited for cases, where a kinematic analysis will be performed, such as clinical scenarios. Kinematic analysis provides a finer level of detail than standard motor impairment scores, such as the Fugl–Meyer Assessment [15] or Action Research Arm Test [16], and has the potential to allow clinicians to distinguish between true recovery and compensatory movements [14, 17]. Using kinematic historical data could also help track a patient’s progress in greater detail [14].

HPE models address the issues highlighted with MoCap systems [6, 9]. These vision-based systems cost significantly less to implement, because they require fewer and less specialized equipment. [18]. Their costs are limited to the sensor (camera) and computational resources necessary for running the HPE model. Vision-based systems can predict joint locations using image data, eliminating the need for markers, meaning that they have lower levels of invasiveness and do not interfere with movements [19, 20]. Unconstrained by a data collection location, HPE models have the ability to easily collect large amounts of data [18, 20]. Data collection and processing is also easier and does not require highly qualified personnel [8].

Around the 2010s, HPE using depth video (red–green–blue-depth, or RGB-D) data and machine learning techniques showed very promising results in terms of potential accuracy and usability [21–23]. As the sophistication and performance of deep learning approaches improved, the HPE field moved away from RGB-D and towards color-based (RGB) approaches, namely, monocular approaches [10, 24]. Although not as prevalent anymore, deep learning-based RGB-D HPE models are still present in the literature. Current methods take advantage of top-performing RGB models, using them to obtain 2D coordinates, and undergo a lifting step to predict the third coordinates for each keypoint. The depth value is taken from the sensor data and assigned to its corresponding joint. In other words, depth is not incorporated in the initial pose estimation step, which is only as good as the 2D pose estimator (and only based on the RGB data) [4, 25, 26].

An important issue faced by 3D RGB HPE models is depth ambiguities, which refers to the fact that multiple different 3D poses may be projected to same points in 2D [10, 27]. Multi-camera markerless MoCap systems can resolve this issue, but, like marker-based MoCap, remain constrained to fixed environments due to the instrumentation required [28]. Alternatively, using depth data from a single camera could help resolve the issue of depth ambiguity, since it provides direct information about the joint’s distance from the camera [4, 28, 29]. Depth data are noisier than RGB, particularly as the distance from the camera increases [29]. Nonetheless, using this type of data could result in a higher prediction accuracy compared to monocular RGB methods [24]. RGB-D cameras have an advantage over multi-camera systems, because they require less setup and do not need camera synchronizations [30]. RGB and depth information are mutually reinforcing, yet there are not many current models that fully take advantage of these complementary sources [29–31].

Another potential limitation of published HPE models in the context of rehabilitation is that they are trained and usually validated using large public data sets, which typically contain data collected from uninjured persons without any disabilities [7, 28]. For 2D HPE, the most popular data sets are COCO [32] and MPII [33]. For 3D HPE, the most widely used are the Human3.6 M [34] and Human-EvaII [35] data sets. Individuals undergoing rehabilitation usually exhibit varying signs or levels of physical impairments that affect how they perform movements [14]. HPE methods have been used to detect neurological conditions or classify if movements are executed correctly [36–42]. Accuracies are reported for persons with injuries in these studies, but errors are not compared to those of uninjured individuals performing the same movements in the same space. Therefore, the impact of motor impairment on HPE model accuracy remains poorly characterized. HPE methods must be validated for their feasibility and accuracy to measure the kinematics of persons with disabilities within the constraints of scalable deployment in healthcare environments.

This work is motivated by the goal to develop a self-contained system that can collect RGB-D data and perform 3D HPE. This device should be low-cost so that it can be easily deployed in different environments. For this reason, we focus on the use of a single camera. It is meant to be used for clinical rehabilitation, meaning that it should be both computationally efficient and accurate. To this end, the present study makes two contributions: (1) we evaluate how incorporating depth data into a lightweight monocular RGB HPE model affects its performance in terms of accuracy and efficiency, in order to guide sensor selection for a scalable solution. (2) We compare HPE model accuracies using data from individuals with and without motor impairments to assess whether accuracy is affected by the presence of disabilities.

## Results

To investigate the impact of depth, we implemented a controlled comparison in which the same models were trained on the same data sets, with and without depth information incorporated. Next, to investigate the impact of motor impairment, we computed HPE accuracy against a marker-based MoCap ground truth, in two groups of participants: one uninjured and the other having experienced a stroke.

### How does depth impact lightweight monocular HPE models?

Two public data sets were selected for this work: CMU Panoptic (CMU) [43] and Human3.6 M (H3.6 M) [34]. These data sets were selected because they are public, large, and provide RGB-D data as well as 3D annotations for joint locations. All ground truth used in this work followed the standard COCO 17-joint skeleton [32] for both the CMU and H3.6 M data. A custom data set (Stroke and Uninjured Depth, or SUD) was also used for training and validating the models. This data set provides RGB-D frames and corresponding 3D ground truth (GT) for six joints on the arms. It includes data from uninjured persons as well as individuals post-stroke. The SUD training data consists of two heathy individuals, its validation data was collected from one heathy individual, and its testing data consists of 12 uninjured participants and 12 individuals post-stroke. The Methods section describes in more detail the SUD data set.

**Fig. 1:**
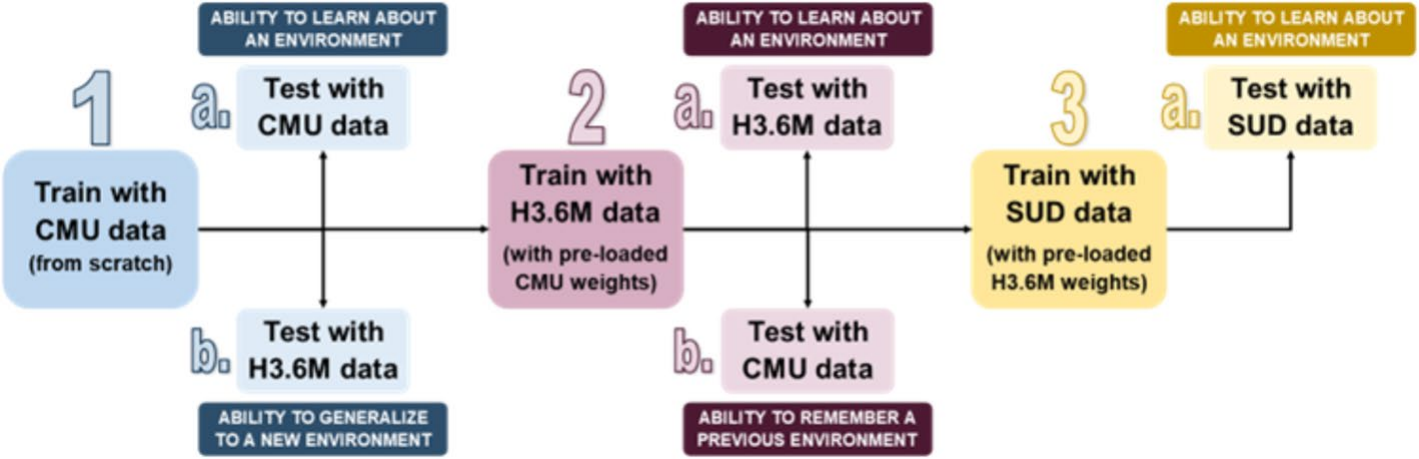
Summary of model training and testing

Figure 1 describes the order in which the models were trained and tested, with regards to the three different data sets. Steps 2 and 3 can be considered fine-tuning for the specific data sets used. Fine-tuning was used rather than training the models from scratch for each data set because of the lower amount of usable data for the Human3.6 M and SUD data sets (~ 250,000 and ~ 25,000 frames, respectively, compared to ~ 2.4 million for CMU).

Dite-HRNet (Dite) [44] and MobileHumanPose (MHP) [45] were used in this work, which perform 2D and 3D HPE, respectively. These models were selected because they use RGB monocular input, are lightweight and open-sourced, and demonstrated the best trade-offs between accuracy and computational efficiency at the time that the literature review was conducted. The Dite and MHP models were modified to include depth data as an input, using three different methods, referred as *CH*, *Cat* and *Fuse*. The CH model has an extra input channel in the first layer of its neural network, whereas the Cat and Fuse methods initially process the RGB and the depth data separately. The Fuse method is more complex than the Cat architecture and incorporates several fusion blocks and additional convolutional layers. The Methods section describes in more detail the three new RGB-D architectures.

Model performance was evaluated in terms of accuracy and computational efficiency. Mean Per Joint Position Error (MPJPE) was recorded for accuracy. MPJPE is reported in pixels for 2D HPE models (MPJPE_pix_) and in millimeters, calculated based on world coordinates, for 3D HPE models (MPJPE_mm_). Floating Point Operations (FLOPs) and frame rates (frames per seconds, or fps) was recorded for efficiency.

#### The effect of depth on accuracy

Table 1 summarizes the accuracies obtained for each Dite and MHP model versions. CMU and H3.6 M results are reported based on MPJPE for all 17 joints, whereas the SUD results are reported for the 6 upper limb joints (right and left shoulders, elbows and wrists) only. For reference, the CMU images have a resolution of 1920 by 1080, H3.6 M images are 1000 by 1000 and SUD images are 1280 by 720 pixels.

**Table 1:** Dite and MHP prediction errors (MPJPE) on testing data

	Trained with CMU (from scratch)		Trained with H3.6 M (weights loaded from CMU training)		Trained with SUD (weights loaded from H3.6 M training)
Tested on	CMU	H3.6M	CMU	H3.6M	SUD
2D HPE using Dite-HRNet (MPJPE_pix_)					
RGB	25.70	114.33^*^	313.07^+^	3.90	11.86 ± 5.33
CH	18.86	94.55^*^	285.92^+^	4.31	11.00 ± 2.80
Cat	22.88	162.70^*^	256.26^+^	5.34	15.27 ± 6.47
Fuse	27.73	146.04^*^	294.54^+^	18.56	16.48 ± 7.96
3D HPE using MobileHumanPose (MPJPE_mm_)					
RGB	12.62	463.95^*^	130.92^+^	79.67	72.52 ± 28.16
CH	12.38	504.32^*^	149.13^+^	72.36	62.79 ± 15.75
Cat	12.27	323.71^*^	108.28^+^	114.06	68.00 ± 14.30
Fuse	13.70	415.17^*^	131.71^+^	156.39	148.52 ± 18.73

^*^ indicates results on environments that the model has not encountered before. ^+^ indicates results obtained when testing on data collected in an environment that was previously used for training (prior to fine-tuning)

**Fig. 2:**
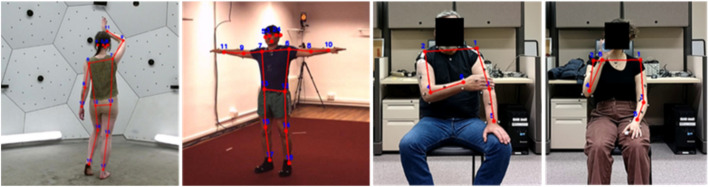
Dite CH model performance (from left to right) on CMU, H3.6 M, SUD (stroke) and SUD (uninjured)

**Fig. 3:**
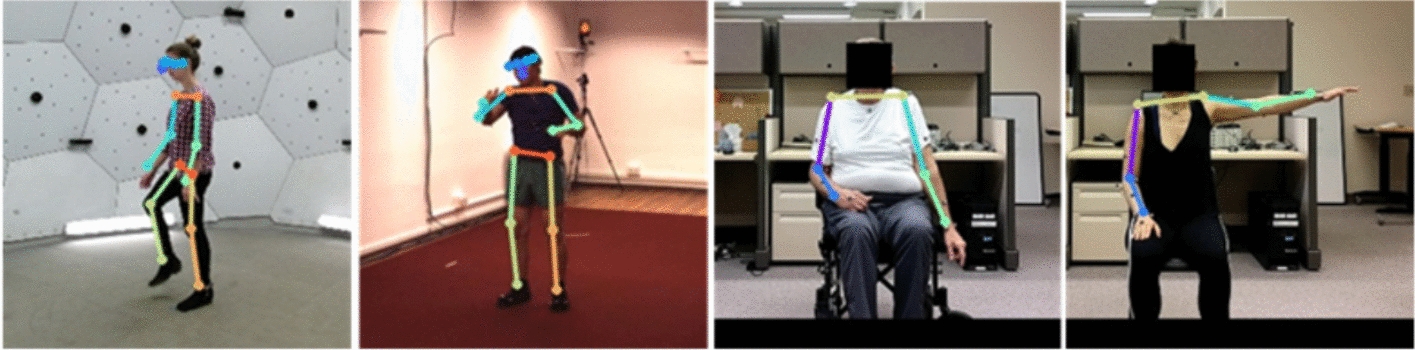
MHP CH model performance (from left to right) on CMU, H3.6 M, SUD (stroke) and SUD (uninjured)

Cells in Table 1 without a superscript highlight the errors obtained when directly testing the models on data collected in the same environment that was provided during training (steps 1a, 2a and 3a in Fig. 1). The CH method consistently predicted poses with lower error than its RGB counterparts for both the Dite and MHP cases, except for one case (Dite when trained and tested on H3.6 M data). Examples of the Dite and MHP CH HPE outputs are shown in Figs. 2 and 3 respectively, on CMU, H3.6 M and SUD data.

Cells in Table 1 indicated with an asterisk highlight the results obtained when testing the model with data collected in an environment that the model has not encountered before. This evaluation was intended to evaluate the model’s generalization abilities, in terms of backgrounds. All models are characterized by substantial MPJPE values, indicating that none do well at predicting poses on images with new backgrounds.

Cells in Table 1 indicated with a plus sign highlight the results obtained when testing the model with data collected in an environment that was previously used for training (prior to fine-tuning). Once again, all models are characterized by very high MPJPE values, meaning that the fine-tuning process negatively affects the model’s ability to perform HPE on images with backgrounds previously encountered during training (from which weights are loaded).

#### The effect of depth on computational efficiency

Table 2 summarizes the efficiency of each Dite and MHP model, based on frame rates and FLOPs.

**Table 2:** Dite and MHP computational efficiency

	FLOPs	Frame Rate (fps)	
		CMU	H3.6M
2D HPE using Dite-HRNet			
RGB	0.90 G	42.8 ± 0.0	58.5 ± 0.5
CH	0.91 G	44.2 ± 2.7	58.0 ± 0.6
Cat	1.79 G	28.3 ± 0.4	33.4 ± 0.2
Fuse	6.17 G	25.3 ± 0.7	31.0 ± 0.1
3D HPE using MobileHumanPose			
RGB	0.97 G	132.3 ± 4.3	180.8 ± 7.4
CH	0.98 G	123.2 ± 4.8	166.2 ± 7.2
Cat	1.61 G	115.5 ± 3.5	154.2 ± 5.2
Fuse	2.88 G	112.9 ± 2.1	146.2 ± 7.5

In terms of FLOPs, there is a clear trend in both the Dite and MHP cases, where the RGB versions have the least FLOPs, closely followed by the CH models. The Cat models come next, followed by the Fuse versions, which are characterized by a considerable jump in FLOPs.

For frame rates, the results vary slightly based on whether the model performs 2D or 3D HPE. In the 2D case (Dite), the CH method results in frame rates that are nearly equal, if not slightly higher, to its RGB counterpart. The Cat and Fuse models, which exhibit similar frame rates to each other, are at around 20 fps slower than their RGB and CH versions. As for the 3D (MHP) case, the frame rates decrease as the model’s complexity increases (as indicated by FLOPs). The MHP CH models are the second fastest out of the four versions explored, after the original RGB models.

### How do physical impairments impact lightweight monocular HPE models?

The custom SUD data set was used for this analysis. Recruited participants belonged to one of the following two groups: uninjured, or with physical impairments to their upper limbs due to a stroke. Participant demographics of the testing data are summarized in Table 3.

**Table 3:** Participant demographic and data information (SUD testing data)

	Uninjured group	Stroke group
Total number of participants	12	12
Age [years]	28.3 ± 12.8	58.3 ± 10.67
Sex	6 males, 6 females	9 males, 3 females
BMI	25.5 ± 5.4	25.6 ± 5.3
Weight [kg]	77.9 ± 17.6	76.7 ± 19.6
Height [m]	1.75 ± 0.09	1.73 ± 0.13
Type of stroke	–	8 ischemic, 4 hemorrhagic
Time since injury [years]	–	3.8 ± 3.4
MAL-14 score	–	36.4 ± 16.4
FMA-UE sections II–IV score*	–	18.6 ± 6.1
Number of frames	8429 ± 942	10,344 ± 2293

^*^The reported FMA-UE (Fugl–Meyer Assessment – Upper Extremity) score is a variation of the full test since only sections II, III and IV of the assessment were performed during the study. The total score is out of 30

RGB-D and optical MoCap data were simultaneously collected while participants performed movements involving their upper limbs only. The MoCap data were used to set the joint location GT, whereas the RGB-D data were fed into the models described in the previous section. Only the upper body was tracked during this study, corresponding to the left and right shoulders, elbows and wrists.

**Fig. 4:**
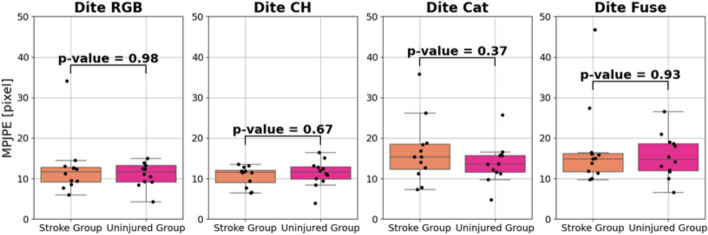
Dite-HRNet model performances on SUD data

**Fig. 5:**
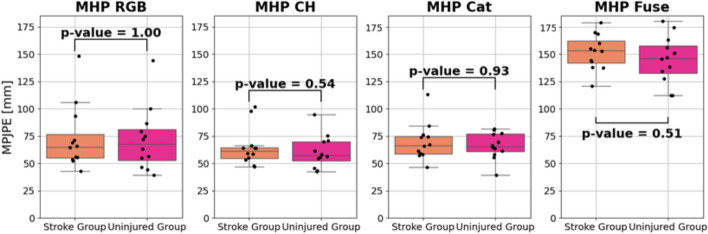
MobileHumanPose model performances on SUD data

The results reported in this section are specific to the models highlighted in step 3 from Fig. 1. MPJPE values were compared between the uninjured and the stroke group. MPJPE box and whisker plots are shown in Figs. 4 and 5 for the Dite and MHP models, respectively. All *p* values are well above the 0.05 threshold, indicating that there is not a statistically significant difference between the MPJPE of the injured and uninjured participant groups.

## Discussion

The main goal of this study was to inform the development of self-contained systems that can collect RGB-D data and perform 3D HPE in scenarios relevant to rehabilitation practice, ranging from hospitals to homes. Such systems should be simple, portable, easy-to-use and low-cost. With the current development and increasing availability of depth cameras, the question arises of how advantageous it is to incorporate depth data into RGB HPE models. The first contribution of this work is to determine how depth data affects a lightweight monocular RGB HPE model in terms of prediction accuracy and computational efficiency.

The CH method consistently provided the most accurate predictions without substantially impacting efficiency in both the Dite-HRNet and MobileHumanPose cases. Depth data was found to be beneficial for HPE accuracy only when incorporated as an early fusion technique. This is the first work that directly compares a 2D RGB model with RGB-D versions, trained using large public HPE data sets. This is also the first work that directly observes the effect of depth data by incorporating them into a 3D RGB HPE model.

Before a HPE model can be incorporated into clinical processes, it must be validated using data collected from individuals with physical impairments. The second contribution of this work is to determine whether the presence of physical impairments affects a HPE model’s prediction accuracy. This is the first work that investigates the effect of physical impairments on a HPE model by directly comparing prediction accuracies between an uninjured and an injured group.

None of the models tested showed a significant difference in joint location prediction accuracies between the uninjured and injured participant groups. This suggests that models trained with public data sets can generalize to persons with physical impairments.

### The impact of depth data on HPE performance

#### Can the model perform HPE in a known environment?

The following discussion refers to the results obtained when the models were directly tested on data collected in the same environment that was provided during training. The advantage of incorporating depth data in terms of HPE accuracy was only consistently apparent when the early fusion (CH) architecture was used.

Looking at the Dite models, when trained (from scratch) and tested with the CMU data, the CH and Cat models had a MPJPE_pix_ of under 25 pixels, making them both more accurate than the original RGB version. The early fusion method (CH) had the lowest MPJPE_pix_, being the only model version with an error under 20 pixels. The models were then trained and tested with the H3.6 M data. Testing revealed that the RGB model became the most accurate, followed very closely by the CH model. Both versions had MPJPE_pix_ values around 4 pixels. Finally, the models were trained with the SUD data. Testing with SUD data revealed that the CH model, once again, had the lowest MPJPE_pix_, standing at an average of 11 pixels. Its standard deviation was also lower than the RGB’s. The Fuse model routinely performed the worst, regardless of the training/testing data.

For the 2D HPE case, the advantage provided by depth data is reduced as more training data are provided. This was demonstrated as the MPJPE_pix_ gap between the RGB and CH model decreased as more training data were used. The CH model was almost 7 pixels more accurate than the RGB when only CMU data were used to train the models. This difference was reduced to less than 1 pixel by the time the SUD data was used for training and testing.

In the case of the MHP models, when trained (from scratch) and tested with the CMU data, all four versions had an MPJPE_mm_ lower than 14 mm. The Cat model had the lowest mean error, followed by the CH, RGB and Fuse models. The RGB, CH and Cat versions had very similar levels of MPJPE_mm_, with only a 0.11-mm difference between the CH and the Cat mean errors. The models were then trained and tested with the H3.6 M data. The CH model was the most accurate, with an MPJPE_mm_ of just under 72.5 mm (over 7 mm lower than the next best-performing model). The Cat and Fuse methods had notably worse accuracies than the RGB version of MobileHumanPose. Finally, the models were trained with SUD data. The CH model consistently outperformed the other model versions, followed by the Cat and RGB architectures.

In contrast to the Dite models, the depth data’s advantage is increased as more training data is provided for the 3D cases. This was demonstrated as the MPJPE_mm_ gap between the RGB and CH model was raised from approximately 0.35 mm (on the CMU data) to 7.31 mm (on the H3.6 M data) to almost 10 mm (on the SUD data). This was anticipated, as depth data were expected to have a stronger positive impact on 3D HPE rather than 2D HPE, given the nature of the task.

In terms of comparing model performances with what was reported in their publications, Dite accuracies cannot be directly compared, because the original model was trained with 2D HPE data sets and used mean average precision as an evaluation metric. As for MHP, the obtained errors, although close (namely, for the RGB and CH versions), are less than 2 cm higher than what was reported in the model’s paper. This difference was expected, since the original model used additional 2D HPE data for training. Qualitative performance in both cases confirms functionality of all models.

#### Can the model generalize to different environments?

Models were tested for their ability to generalize to new environments in step 1b (Fig. 1). None of the Dite and MHP versions of the models were able to adequately perform HPE on images with a new background (not encountered during training). Performances were characterized by substantial errors. The MPJPE performances for these cases are not compared in terms of relative performances because of low levels of prediction quality. Nevertheless, this does not affect our conclusion that depth data improves prediction accuracy when tested on data collected in a familiar environment.

This poor performance is hypothesized to be caused by the low levels of background variability during training. Both the CMU and H3.6 M data sets were collected in controlled environments with relatively plain backgrounds that were common to all frames. Referring to Figs. 2 and 3, the CMU data was collected in an environment with white walls, a concrete-colored floor and no hard corners, whereas the H3.6 M data’s background is characterized by warmer beige walls, a dark red carpet, and a corner at which a camera stand is visible.

The two most popular 2D HPE data sets used for training and testing newly proposed 2D HPE models are the COCO [32] and the MPII [33] data sets. COCO contains images collected from Flickr and is very rich in terms of contextual information [32]. MPII [33] is composed of images taken from various YouTube videos. Due to the rich contextual information present in the COCO and MPII data sets, it is not surprising that models trained with their data do not experience environmental generalization issues. HPE testing results on either data set are indicative of a model’s environmental generalization capabilities, as the images used for testing have different backgrounds that those used for training. In addition, 2D HPE models are commonly evaluated in papers on specific, privately collected data. For instance, OpenPose [46], which reports results on both COCO and MPII, has no problem performing well on new images with different backgrounds, as demonstrated by its popularity for a wide range of uses in the literature [47, 48].

On the other hand, the performance of 3D HPE is limited by the current lack of large and public in-the-wild-data sets [28, 49]. Methods have more difficulties generalizing to images collected in-the-wild, because most public 3D HPE data sets use optical MoCap systems to set the joint location GT, meaning that images are collected in a controlled and relatively simple environment [27].

Data augmentation techniques were incorporated into our Dite and MHP models as an attempt to improve generalization abilities, but this was not enough to provide different contextual information as found in images with different backgrounds. The environmental generalization issues experienced by the models in this work could be addressed using more diverse training data in terms of backgrounds. Unfortunately, a large non-synthetic in-the-wild 3D RGB-D HPE data set does not exist yet.

The models were able to learn about new environments when that information was incorporated into the training set (training with H3.6 M data after pre-training on the CMU data). The advantage of doing so was evident when comparing testing accuracy on H3.6 M before and after the fine-tuning. Using H3.6 M as additional training data greatly improved the accuracy on the data set’s test set. For the Dite cases, the MPJPE_pix_ decreased from approximately 100 to 5 (excluding the Fuse method). As for MHP, the MPJPE_mm_ went from over 460 to under 80 for the RGB and CH versions of the model.

The fine-tuning process had a drawback: prediction accuracy on the CMU test data was considerably reduced after training with the H3.6 M data, even if the weights obtained during training with CMU data were used (step 2b in Fig. 1). This behavior can be explained using the phenomenon of catastrophic forgetting. Catastrophic forgetting occurs in neural networks when a model forgets previously learned optimal weights that worked well for a specific task (or data set) and learns a new set of optimal weights specific to a new task [50–52]. A variety of methods have been proposed to address catastrophic forgetting and could be explored for RGB-D HPE in the future [51, 53–55].

#### How is the model’s computational efficiency affected?

The architecture resulting in the least FLOPs was the RGB (original) for both the Dite and MHP models, with the CH (early fusion) versions being close seconds. The intermediate fusion methods (Cat and Fuse) had the highest number of FLOPs. These results are consistent with the literature: early fusion techniques are simpler than intermediate ones and require fewer calculations.

Overall, adding depth as an input channel (CH) did not considerably affect the model’s speed (frame rates). The CH method either resulted in similar frame rates to their RGB counterparts or the second highest. Cat and Fuse led to more pronounced reductions in frame rate. The difference in inference speeds between the CMU and H3.6 M data is hypothesized to be caused by different image resolutions. The CMU data (1920 × 1080) has a higher resolution and is almost double the size of the H3.6 M (1000 × 1000) data, which could contribute to slightly higher pre-processing times.

All models, regardless of the data, had testing frame rates higher than 24 fps, which is close to the speed at which frames are collected in a standard camera (30 fps), suggesting inference time capabilities close to real-time as long as testing is performed on a GPU-equipped machine. The MHP models specifically reached frame rates higher than 100 fps, indicating very promising potential for real-time HPE applications, and the possibility of achieving acceptable frame rates on hardware with lower capabilities.

#### Final recommendations—should depth data be incorporated into HPE?

Considering the effects of depth discussed in the previous sections, we determined that the CH method, regardless of whether it is incorporated into 2D or 3D HPE, results in the best trade-off between accuracy and computational efficiency. This method yielded the most accurate predicted poses (by having the lowest MPJPE) with a negligible increase in FLOPs and minimal impact on frame rates compared to the RGB version of the models.

### The impact of physical impairments on HPE accuracy

We hypothesized that there would be no significant difference in accuracy between the two groups recruited during this study. Although impaired participants may move differently than uninjured individuals, participants with a stroke were not expected to generate poses that were drastically different from the ones executed by persons without injuries. Their movements were characterized by slower speeds and compensatory strategies when needed.

Even though they were trained with data from persons without injuries (from the CMU, H3.6 M and SUD data), all the Dite and MHP models were able to generalize to persons with physical impairments, with no statistically significant differences found in HPE accuracies. This suggests that HPE models trained and validated using large public data sets collected from uninjured individuals are expected to have comparable accuracies in a rehabilitation context. These models should be ready for use in clinical scenarios to the extent that the accuracies that they achieve are suitable for a given medical application.

### Limitations and future work

Data availability was the biggest limitation for this study. There is a restricted number of public 3D HPE data sets that provide both RGB and depth data, which was necessary for us to implement a direct comparison of RGB and RGB-D models. Of the few that are available, none consist of images collected in-the-wild, unlike 2D RGB HPE data sets. Using a more varied data set (with different backgrounds and environments) may result in better RGB-D generalization abilities or bridge the accuracy gap between the RGB and RGB-D models. The increasing availability of RGB-D sensors make in-the-wild data collection more accessible. Exploring the effect of depth on models trained with richer data sets would be interesting for future work.

It is important to reiterate that the purpose of this work was not to surpass the accuracies of the current state of the art for lightweight monocular RGB HPE models. Rather, our goal was to determine how adding depth as an input affects a model’s performance in terms of accuracy and computational efficiency. The models presented in this work do not surpass the state-of-the-art accuracies or speeds. Although performances cannot be directly compared due to slightly different protocols used for training, SRNet [56] achieved an MPJPE_mm_ of 36.6 mm on the H3.6 M data set. Another model, [57], reported an MPJPE_mm_ of 52.7 mm on the same data set. The models presented in this work are at least 20 mm less accurate than the current monocular RGB HPE state-of-the-art on H3.6 M. Model selection in this study was guided by a balance of accuracy, computational efficiency, and availability for modification to implement the depth fusion strategies. Given the rapid pace of the development in deep learning-based HPE, future work should focus on exploring the effect of depth on newer methods. The work presented here suggests that, although the HPE models investigated may not yet achieve accuracies sufficient for clinical use, RGB-D models trained on public HPE data sets (collected from healthy and uninjured individuals) have potential for applications in clinical environments.

While joint location estimates are an essential key step and the focus of the present work, applying kinematic analysis to answer clinical questions is likely to require additional inverse kinematic steps involving biomechanical models to estimate precise joint angles [58]. It should also be noted that SUD ground truth based on markers corresponds to slightly different joint positions that those in the CMU and H3.6 M data sets (e.g., center of the shoulder joint). While this difference may have contributed slightly to error values, it is expected to be partially mitigated by the re-training performed on SUD data.

Investigating RGB-D HPE performance on low-resource settings, such as microcontrollers or microprocessors, will be warranted, since real-time capabilities of the models under these conditions is of interest for use in varied clinical or home settings.

The conclusions from this work regarding motor impairment are based only on individuals with stroke performing upper limb movements and on two HPE models (Dite and MHP) explored. The results were promising and seem to indicate that HPE accuracy is not affected by the presence of physical impairments. Future work should focus on testing different HPE models and recruiting a varied group of participants with different types of injuries, not only limited to the upper extremities, as well as different demographics and individuals with amputations or malformations.

Multi-person scenarios should also be investigated, since rehabilitation sessions typically involve a clinician guiding a patient.

Many rehabilitation movements involve interacting with objects, which can occlude a person’s body. The data collected during the study includes self-occlusion, but only one interaction with an object (a water bottle) was recorded. In the future, more data capturing interactions with objects should be collected. Some participants from the injured groups used walking aids, such as canes, but this was not captured in the collected data. Assistive devices can cause occlusions that are normally not encountered in uninjured individuals. It would be worthwhile to investigate how different aids affect a HPE model’s accuracy, and whether using a RGB-D model is more robust for these cases.

## Conclusion

The results of this work demonstrated that adding depth as an input to HPE models led to improvements in accuracy compared to RGB models with a minimal cost to computational efficiency, when implemented using a simple early fusion technique. The model accuracies were consistent regardless of whether data from injured or uninjured individuals was used for testing. Integrating deep learning HPE models into rehabilitation processes could reshape procedures in a way that would benefit patients individually and systemically. On a patient level, it can lead to more precise ongoing motor assessment and support personalized interventions. On a systemic level, HPE can provide for the first time a scalable approach to capturing data about recovery trajectories in rehabilitation, playing a key role in improving the evidence base for rehabilitation interventions.

## Methods

### The impact of depth data on HPE performance

The following steps were followed to implement a controlled comparison of RGB versus RGB-D models: (1) Select a 3D RGB-D HPE data set. (2) Select a 2D and a 3D lightweight monocular RGB HPE model. (3) Modify the HPE models to include depth as an input. (4) Train the RGB models and their RGB-D versions, using the same protocol on the same data set. (5) Compare the models’ performance (accuracy and computational efficiency).

#### Data set selection

The CMU Panoptic (CMU) [43] and Human3.6 M (H3.6 M) [34] data sets were selected for this work. A private, custom data set (SUD) was also used for training and validating the models.

No standard training/validation/testing data split protocol was found for the CMU Panoptic data set, so a unique protocol was defined. The *3D PointCloud Data set Version 1* was downloaded from the *KinopticStudio Github* page [59]. Only the trials with RGB-D data and single-person scenarios were kept. Data was split as an attempt to follow an 80/10/10 split, as summarized in Table 4 [60].

**Table 4:** CMU Panoptic (Kinoptic) data split

	Training set	Validation set	Testing set	Total
Trial names	161029_flute1	170915_office1	170307_dance5	
	161029_piano2, 3, 4	171026_pose2	171204_pose6	
	170407_office2	171204_pose3		
	171026_cello3			
	171026_pose1, 3			
	171204_pose1, 2, 4, 5			
# Frames	1 804 140	287 400	326 980	2 411 860
% of Total	74.6%	11.9%	13.5%	100%

A variation of the H3.6 M protocol 1 was used for the data split. The original protocol assigns subjects 1, 5, 6, 7, and 8 to the training and subject 9 and 11 for validation [61]. Since testing data is not available for download, subject 9 was used for validation and subject 11 for testing.

The SUD data is composed of videos from 15 different uninjured individuals, and 12 post-stroke patients. Data from the first three uninjured participants was used to train and validate the models (participants 1 and 2 for training, and participant 3 for validation). Data from the remaining 12 uninjured participants and the 12 stroke participants was used for testing.

#### Pre-processing

Human bounding box locations for each image were generated using YoloV7 [62] and incorporated into the GT files. All GT skeleton annotations were modified to respect the standard COCO 17-joint format [32]. Raw depth data was cleaned using a dilation filter. The OpenCV *registerDepth* function [63] was used to register the depth to the RGB data. Depth registering refers to the data transformation process of aligning depth images to RGB images, both in terms of resolution and field of view. Resulting RGB-D files were saved as 4-channel images. Provided timestamps were used to match each GT skeleton annotation to its closest RGB-D frame.

The *panutils.projectPoints* function [64] was used to convert the 3D GT to its 2D location for each view (project 3D points to 2D locations in the corresponding camera frames). This function uses 3D GT coordinates and camera parameters as an input: intrinsic, which consists of the camera’s optical center and its focal length, and extrinsic, which is a representation of where the camera is located in the 3D world. The H3.6 M depth camera parameters were not provided in the original data set. Extrinsic parameters were approximated by copying camera 2’s parameters, which were approximated to be the same, since the depth sensor was placed right above it. The depth camera’s intrinsic parameters were taken from [65]. Only the data from camera 2 was used, because it was the only one that had corresponding depth data.

#### HPE model selection

Temporal HPE models were not considered because of the lightweight requirement. Multi-view models were also discarded as options, because the type of setup required is not compatible with the study focus on a low-cost, portable device for data collection in different environments. Multi-view setups are considerably more expensive and require more complex models [28]. Most lightweight models are monocular due to their lower complexities and low-costs [6]. Thus, the search was limited to monocular RGB models. With these constraints in mind, the Dite-HRNet [44] and MobileHumanPose [45] models were selected for this work. MobileHumanPose was modified to use a sampling ratio of 1 and to predict COCO keypoint locations instead of its original 17.

Dite-HRNet performs 2D HPE, and outputs a list of joint locations in image coordinates (in pixels) for each frame. MobileHumanPose perform 3D HPE, and converts its outputs to joint locations in world coordinates (in millimeters).

#### HPE model architecture modification

Early fusion techniques are appropriate for situations, where the different data types share the same resolution and sampling rate [66]. Intermediate techniques fuse data at the feature level and are the most popular type in the literature [67]. Because of this, an early fusion (CH) and two intermediate fusion (Cat and Fuse) techniques were investigated in this work. Late fusion techniques were not explored, because they do not align with the lightweight model requirement.

**Fig. 6:**
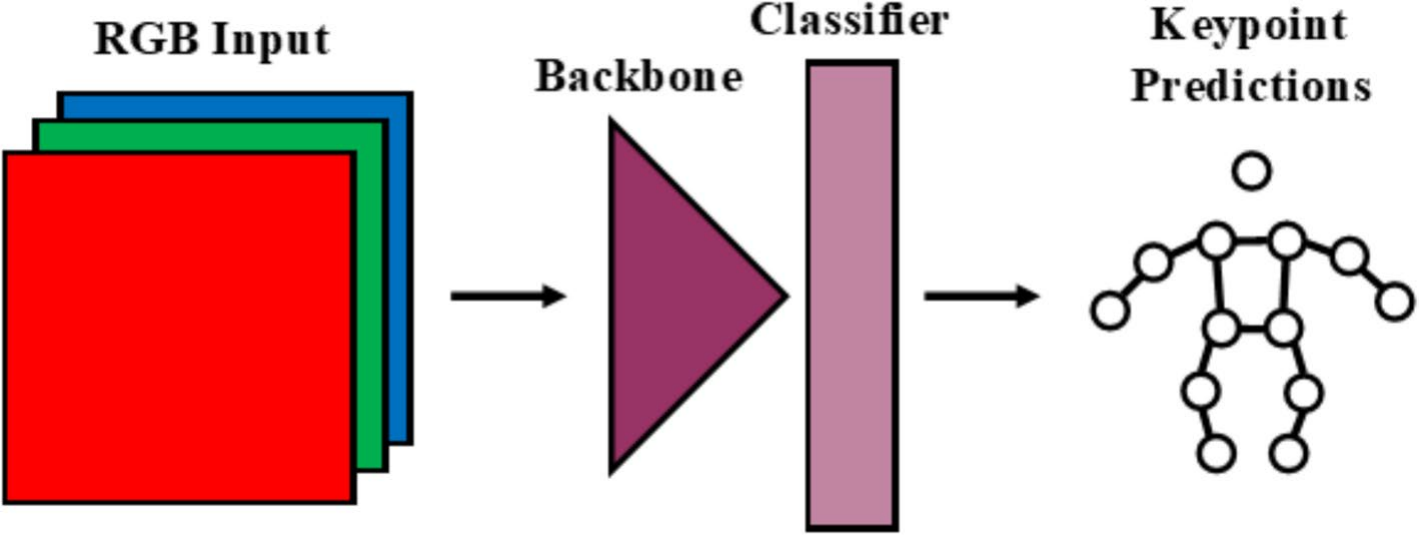
RGB architecture

**Fig. 7:**
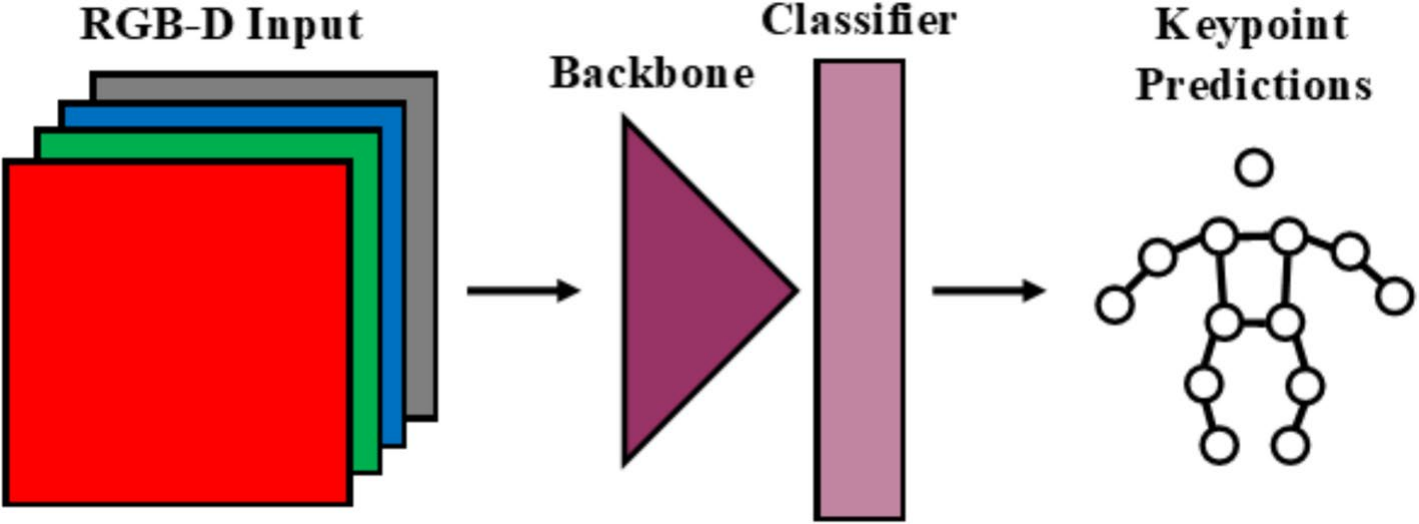
RGB-D CH architecture

**Fig. 8:**
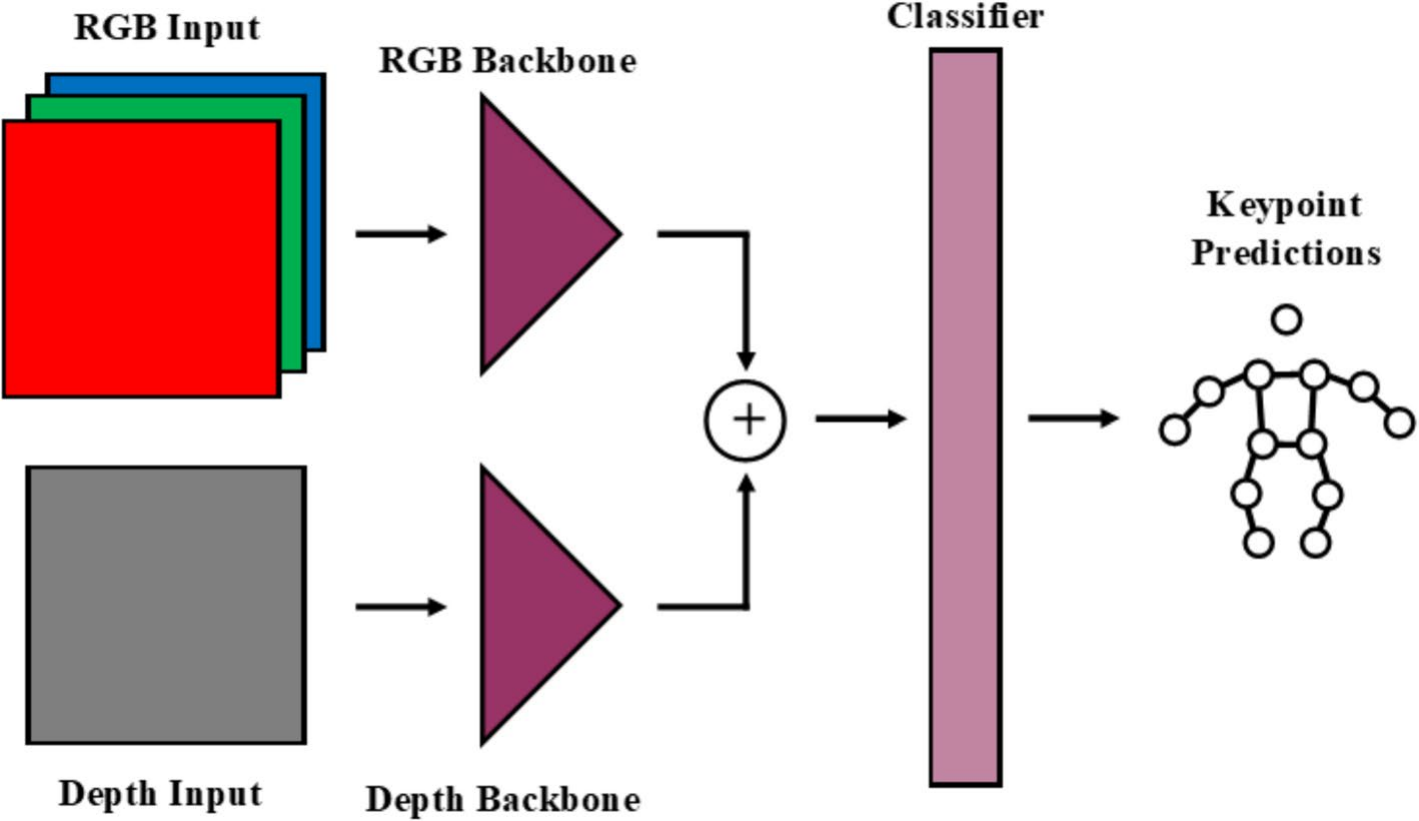
RGB-D Cat architecture

**Fig. 9:**
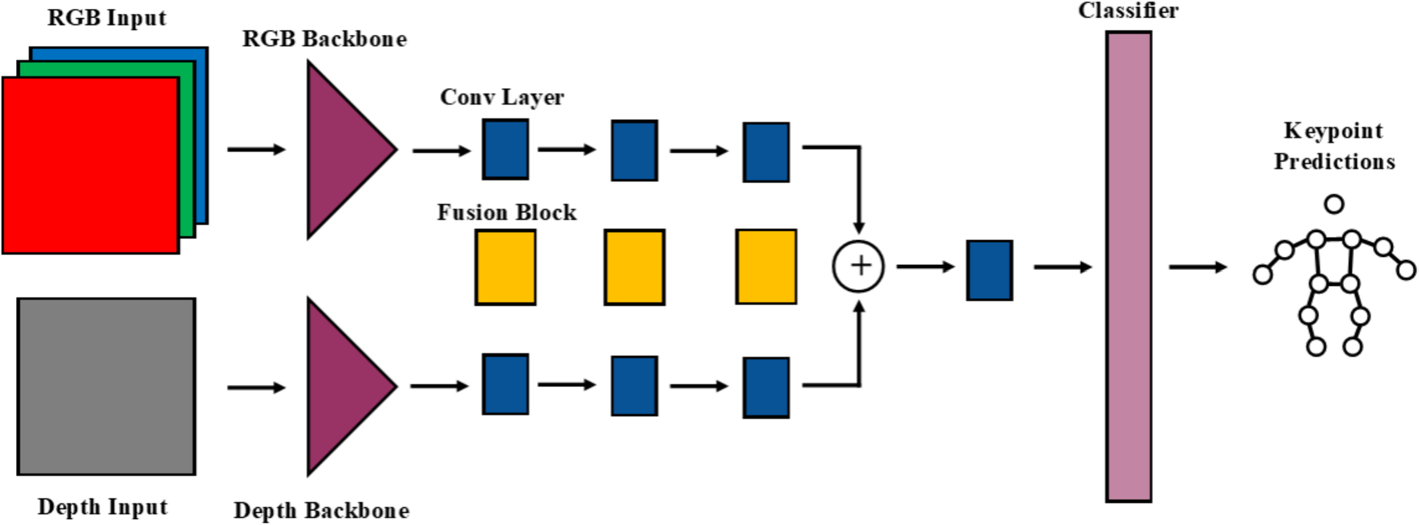
RGB-D Fuse architecture

In both the Dite and MHP models, the RGB data are fed into a backbone (with three input channels), which extracts useful features. These features are fed into a classifier, which determines the location of the joints. A simplification of this architecture is shown in Fig. 6. The CH method added an input channel to the model’s backbone to accommodate for a four-channel input (RGB-D) instead of the regular three channels (Fig. 7). The Cat method uses identical but separate backbones for the color (RGB) and depth (D) data and concatenates extracted features before feeding it into the classifier (Fig. 8). The Fuse method also uses separate backbones for the color and depth data, and incorporates fusion blocks, introduced in [68] (Fig. 9). The fusion blocks concatenate the RGB data, depth data and their dot product before feeding them through convolution and ReLU layers.

The CH method, being an early fusion technique, was selected, since it is the simplest fusion method. It is expected to perform well for this case, since the RGB and depth data shares the same resolution and sampling rate. Intermediate fusion has the potential of learning more complex relationships between the RGB and the depth data, but at the risk of being more computationally intensive. Intermediate fusion techniques were investigated due to their popularity in the literature. The Cat method is the simplest intermediate fusion technique that can be applied, whereas the fusion method is more sophisticated. The Fuse method was specifically selected because it was developed and presented as an RGB-D fusion architecture.

#### Model training

Hyperparameter tuning was not used for any of the models to enable a direct comparison between the RGB models and their RGB-D equivalents. Original parameter values from the model *Github* pages were used (see Table 5).

**Table 5:** Model training parameters

	Dite-HRNet	MobileHumanPose
Epochs	10*	25
Learning rate	1e-3	1e-3 (decreased by a factor of 10 after the 17th and 25th epoch)
Batch size	16	64
Input shape	384 × 288	256 × 256
Tensor normalization	Mean: (0.485, 0.456, 0.406, 0.500) Standard deviation: (0.229, 0.224, 0.225, 0.366)	Mean: (0.485, 0.456, 0.406, 0.500) Standard deviation: (0.229, 0.224, 0.225, 0.366)
Number of workers	2	40
Number of GPUs	4	4

^*^The original Dite-HRNet model was trained with 270 epochs, which was not feasible in this study. Pilot training revealed that, when training Dite-HRNet with the full CMU data set, the model was done most of its learning within the first epoch. Therefore, a standard 10 epochs of training was set for this model

#### Model performance evaluation

Models were compared on testing data based on MPJPE for accuracy and inference speed for efficiency, as well as their FLOPs. MPJPE was calculated using Eq. 1 [28], which is the Euclidean distance between the predicted joint location and its ground truth. In Eq. 1, *f* represents the frame number, *S* is its corresponding skeleton, *N*_*S*_ is the total number of joints in the skeleton, ${P}_{f,S}^{\left(f\right)}$(i) is the predicted location of joint *i* and ${P}_{gt, S}^{\left(f\right)}\left(i\right)$ is its corresponding GT location [28]. Dite FLOPs were calculated using built-in *MMPose* functions [69]. MHP FLOPs were calculated using a mix of code provided with the model and the *calflops* library [70]. This library had to be used, because the originally provided code did not support some of the layers in the models.

1 $$E_{MPJPE} \left( {f, S} \right) = \frac{1}{{N_{S} }}\mathop \sum \limits_{i = 1}^{{N_{S} }} || P_{f,S}^{\left( f \right)} \left( i \right) - P_{gt, S}^{\left( f \right)} \left( i \right)||_{2}$$

The best epoch for each model was identified based on its performance on the validation data and used to test the model. All models were tested on the same computer with 1 GPU (NVIDIA GeForce GTX1080 Ti), so frame rates can be directly compared. Reported frame rates were obtained after testing 28,002 images of each data set (CMU and H3.6 M).

### The impact of physical impairments on HPE accuracy

The following steps were followed to evaluate the impact of motor impairment on HPE accuracy: (1) collect RGB-D as well as marker-based MoCap data from uninjured participants and participants who had experienced a stroke. (2) Feed the RGB-D data into the 2D and 3D pose estimation models. Evaluate pose estimation accuracy using the MoCap ground truth. (3) Compare accuracies between the injured and uninjured groups.

#### SUD data collection

The study recruited individuals pertaining to one of the following groups: uninjured, free of any known medical condition impacting their muscular system (15 participants) or with upper limb impairment(s) due to a stroke (12 participants). Participants from the stroke group had to have scores between 14 and 56 (average score between 1 and 4 on a 6-point scale) on the self-reported Motor Activity Log 14 (MAL-14) [71]. Participants were simultaneously recorded with MoCap system and an RGB-D camera, while they performed movements involving their arms. The MoCap data were used as joint location GT and the depth video as input for the HPE models.

Data were collected as the participants executed the following movements: FMA-UE sections II, III and IV (hand from contralateral knee to ipsilateral ear, hand to lumbar spine, shoulder flexion, pronation–supination with elbow at 90°, shoulder abduction, shoulder flexion and pronation–supination with elbow at 0°) [72], shrug shoulders, wave, open a water bottle, drink from it and close it, touch wrists, elbows and shoulders, stretch their arms out, make circular arm motions and punch the air. Participants were asked to perform movements beyond those included in the FMA-UE, based on their interpretation of the verbal instructions. All movements were performed for each side (right and left) individually.

**Fig. 10:**
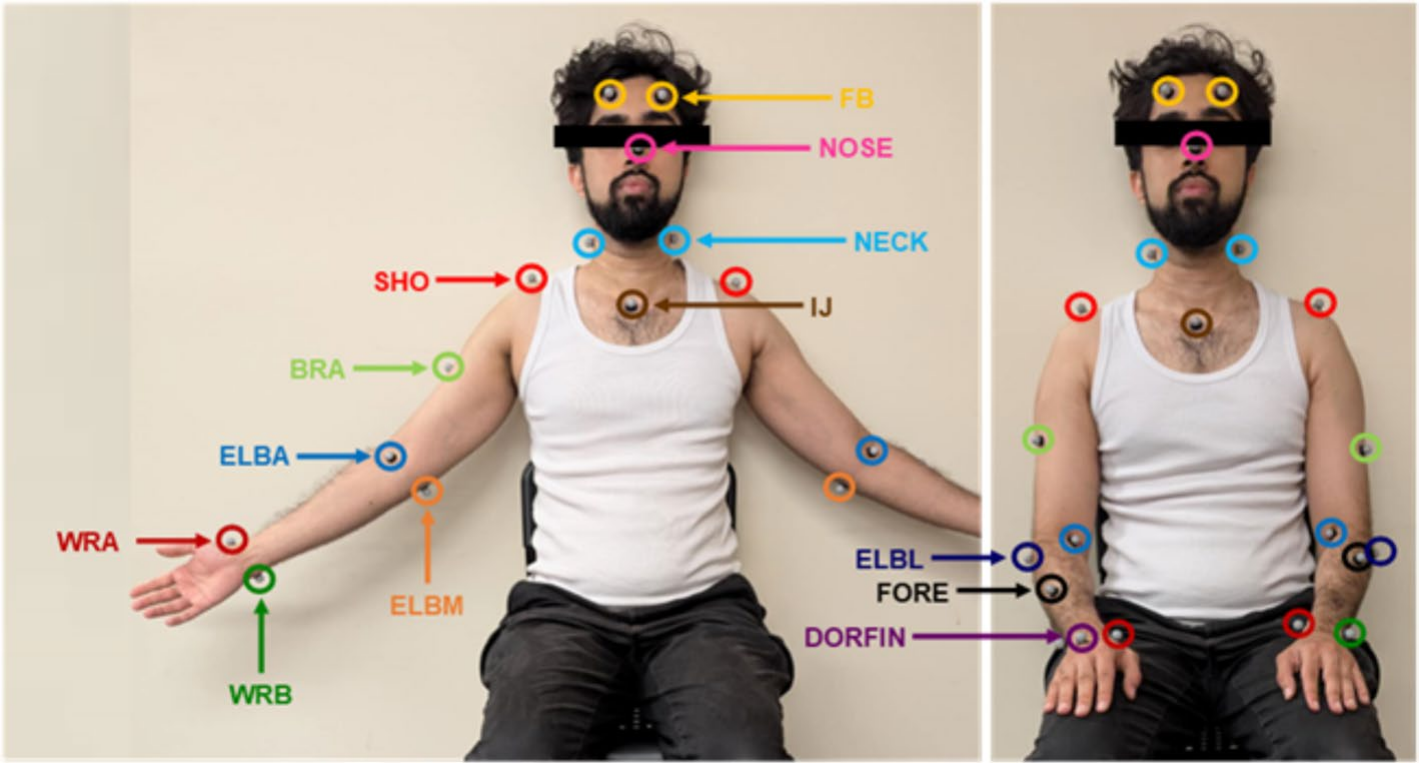
Marker placement on participants

The MoCap data was collected at 90 Hz using a Vicon [73] system, consisting of 8 cameras surrounding the participants. The system setup allowed for the recording of a rectangular area of 4.9 by 3.6 m, with the participant seated at its center. 23 reflective markers were placed onto each participant’s upper limbs. Table 6 lists the marker names and their corresponding anatomical locations. Figure 10 shows the markers on a participant. The marker configuration was based on other upper limb MoCap studies, such as those in [74] and [75]. The MoCap data was labelled and gaps were filled using Vicon built-in pipelines. The marker trajectories were filtered with the built-in Woltring filter before exporting the data.

**Table 6:** Marker names and anatomical locations [74]

Marker Name	Anatomical Location
FB	Frontal bone of the head, above the eye
NOSE	Tip of the nose
NECK	Middle of the posterior triangle of the neck
IJ	Deepest point of incisura jugularis
SHO	Most dorsal point on the acromioclavicular joint
BRA	Most proximal point of the brachialis
ELBA	Antecubital fossa
ELBL	Most caudal point on lateral epicondyle
ELBM	Most caudal point on medial epicondyle
FORE	Midpoint of the posterior forearm
WRA	Most caudal–lateral point on the radial styloid
WRB	Most caudal–medial point on the ulnar styloid
DORFIN	Distal end of the 3rd metacarpal phalanx

Participants were asked to touch their nose before executing each movement. The MoCap data was manually synced to the collected RGB-D data by identifying the frame at which the participant touched their nose, and the last frame at which the movement was completed. This manual synchronization method was used due to its simplicity, and its quality was thoroughly checked through visual inspections.

Depth video data were collected using a stereo RGB-D camera (OAK-D S2, Luxonis, Denver, CO, USA). The camera was placed 2.2 m away from the participant, directly in front of them. This location was kept constant for every participant. The camera’s extrinsic parameters were obtained using the OpenCV chessboard calibration procedure [76]. Color and disparity videos were collected at 30 fps at a resolution of 720p. Disparity was recorded (the OAK camera is not able to directly record depth), and then used to compute depth values. Depth sensors that use stereo vision technology, as the camera used in this study, use disparity to calculate depth values using Eq. 2 below, where B is the baseline distance between the two stereo cameras and f is the focal length [77]. The RGB-D files were created the same way as the CMU and H3.6 M data:

2 $${\varvec{Depth}} = \frac{{{\varvec{B}}\cdot{\varvec{f}}}}{{{\varvec{Disparity}}}}$$

**Fig. 11:**
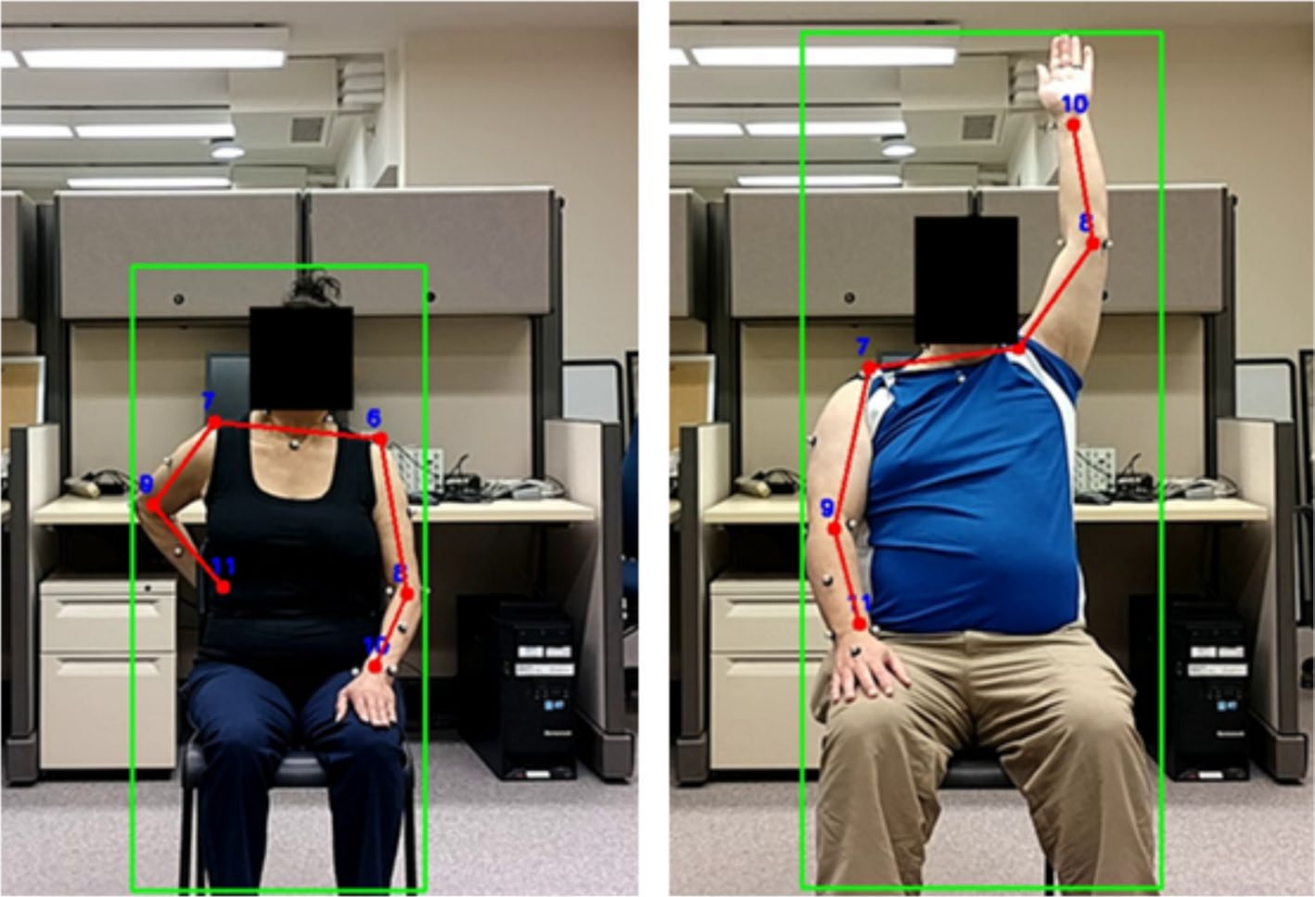
Training data example from the two uninjured participants. The GT skeleton is drawn in red, and the bounding box in green

The GT for the six arm keypoints was set based on Table 7. MPJPE values were calculated only for these keypoints. Other body landmarks were not included in the MPJPE, because only the arms were tracked during this study. Figure 11 shows an example of the data used to train the models (from the two uninjured participants).

**Table 7:** Defining the GT joint locations

GT location (right and left sides)	Location based on markers
Wrist	Mid-point between WRA and WRB
Elbow	Mid-point between ELBA, ELBL and ELBM
Shoulder	Shoulder (SHO) marker location

#### Statistical analysis

The Mann–Whitney *U *test was used determine if there was a statistically significant difference in HPE accuracies between the recruited uninjured and stroke groups. A non-parametric test was used due to the lack of normal distribution in some samples, after testing for normality using the Shapiro–Wilk test.

## Data Availability

The code for the models described is available at https://github.com/ANSLab-UHN/rgbd-pe. The SUD data include identifiable video data and cannot be shared, per the terms of our institutional approvals and informed consent.
